# Thermoplastic Zinc-Infused Polymer for Chairside Socket Seal Abutments Enhances Antimicrobial and Tissue-Integrative Properties

**DOI:** 10.3390/antibiotics14050441

**Published:** 2025-04-27

**Authors:** Wannes Van Holm, Katleen Vandamme, Jill Hadisurya, Ferda Pamuk, Naiera Zayed, Merve Kübra Aktan, Annabel Braem, Andy Temmerman, Wim Teughels

**Affiliations:** 1 KU Leuven, Department of Oral Health Sciences, Research Unit Periodontology and Oral Microbiology (P&OM), B-3000 Leuven, Belgium; wannes.vanholm@kuleuven.be (W.V.H.); naiera.zayed@kuleuven.be (N.Z.);; 2 KU Leuven, Department of Materials Engineering, Biomaterials and Tissue Engineering Research Group, B-3001 Leuven, Belgium

**Keywords:** peri-implantitis, soft-tissue engineering, zinc, wound healing, biocompatibility, healing abutment

## Abstract

**Background/Objectives**: The essential trace element zinc (Zn) has a pivotal role in wound healing and can show antibacterial activity, but its application in oral implant materials is underexplored. Customized healing abutments can modulate the peri-implant tissue health when appropriate bioactive materials promoting mucosal healing are used. The present study investigated a novel Zn-containing polymer for its potential in soft-tissue engineering applications. **Methods**: Four traditional materials—titanium, glass ionomer, a composite, and the novel Zn-containing polymer—were tested in vitro for bacterial growth using a multispecies oral bacterial model compared to hydroxyapatite. The biocompatibility of the materials was also evaluated by evaluating the adhesion, proliferation, and cytotoxicity of human oral keratinocytes (HOK-18A) onto these materials, compared to tissue culture plastic. **Results**: The Zn-containing polymer exhibited a significantly lower biofilm formation compared to conventional materials as it was composed of less pathogenic bacteria. The Zn-containing material also demonstrated a superior biocompatibility towards HOK-18A, approximating the adhesion and proliferation of the keratinocytes to optimal tissue culture conditions. Moreover, these properties did not seem to degrade and were maintained over a period of 31 days. The cytotoxicity assessment revealed no significant reduction in metabolic activity for any material. **Conclusions**: This study highlights the potential of the novel Zn-containing polymer in soft-tissue engineering, owing to its antimicrobial and biocompatible assets. These properties, combined with the ease of chairside modeling, position the material as a promising alternative for creating customized healing abutments. Further research is needed to explore its mechanism of wound healing modulation and its clinical performance.

## 1. Introduction

The success of implant-supported prosthetic treatments depends not only on the esthetic outcome of the final restoration but also on the health and appearance of the adjacent soft tissues [1]. To achieve an optimal soft tissue appearance, creating a natural emergence profile is essential. This process is particularly challenging during the healing phase after implant placement [2]. Standardized prefabricated titanium healing abutments have traditionally been used for shaping the peri-implant soft tissues. However, their inability to replicate the natural emergence profile of teeth has resulted in the development of custom healing abutments [3,4,5]. Originally, this involved attaching a temporary cylinder to the implant fixture and manually shaping it with composite resin to match the patient’s tooth unique anatomy [6]. Commonly referred to as socket seal abutments (SSAs), these chairside custom abutments provide a quick, cost-effective solution for sealing the socket and preserving the soft tissue morphology, definitely in cases where the implant is placed immediately after extraction [7]. Chairside fabrication offers a fast and more practical approach for creating custom healing abutments.

By using digital workflows and leveraging CAD/CAM technology, optimal customization can be ensured, creating custom healing abutments in Ti, PEEK, or Zirconium. While effective for complex cases, indirect fabrication often requires detailed planning and can be resource-intensive compared to chairside methods performed directly during surgery.

When it comes to the material used to create chairside customized healing abutments, concerns about the cytotoxic effects of resin composite materials on gingival cells remain a critical consideration. Certain resins can negatively affect gingival fibroblasts and epithelial cells [8,9]. Moreover, specific resin components like UDMA and TEGDMA have been identified as potentially harmful [10]. Given the importance of epithelial attachment in safeguarding underlying tissues from the harmful effects of bacteria, as well as maintaining peri-implant tissue health and stability [11,12,13,14], material selection for these abutments must be approached with caution.

To achieve ideal outcomes, materials used for chairside custom healing abutments must possess specific biological properties. They should promote the adhesion and proliferation of fibroblasts and epithelial cells, ensuring a secure tissue seal, facilitating the maturation of peri-implant mucosa, and maintaining the peri-implant tissue architecture, including papilla height and mucosal margins [15,16]. The material surface should also prevent excessive bacterial adhesion, reducing the risk of inflammation and infection [17]. These properties are critical to achieving stable and healthy peri-implant tissues and long-term functional success. Last but, from a clinician’s standpoint, definitely not least, the material should be easily applicable from a clinical point of view. This means that it should be easily adherable to the Ti temporary cylinder and easily adjustable to achieve the desired form.

Advances in soft-tissue engineering have introduced innovative biomaterials designed to promote tissue regeneration by mimicking natural properties. Zinc (Zn)-containing materials, in particular, have demonstrated promising effects, such as enhancing cell growth, promoting epithelialization, and reducing bacterial colonization [18,19,20]. Clinical evidence supports the advantages of Zn-embedded polymer stents, including faster healing, reduced pain, and minimized bleeding, highlighting their potential for oral tissue applications [21]. These findings suggest that certain Zn-based polymers could play a valuable role in shaping the emergence profile of peri-implant tissues.

While titanium and ceramic abutments are known for their biocompatibility and healing support [22,23,24,25], chairside-fabricated custom abutments using materials such as composite, glass ionomer, or Zn-containing polymers remain underexplored for their biological safety and efficacy [23,26,27].

To address this gap, the present study investigates a novel thermoplastic Zn-containing polymer designed for creating chairside custom healing abutments. This material’s thermoplastic properties allow for precise adaptation and ease of use during chairside fabrication, while its biocompatibility offers potential advantages for soft tissue healing. The study evaluates bacterial adhesion, cell growth, and biocompatibility of this innovative material in comparison to commonly used options (titanium and composite), glass ionomer, and controls such as hydroxyapatite and tissue culture plates. Glass ionomer was also explored as a potential material, despite its limited application in this context. While its mechanical strength may be lower compared to composites [28], its more favorable biocompatibility makes it an interesting material for investigation in peri-implant healing [29].

By assessing these properties, this research aims to determine whether thermoplastic Zn-containing polymers can provide a reliable, biocompatible, and cost-efficient solution for both the chairside and indirect fabrication of custom healing abutments, ultimately improving patient outcomes in implant dentistry.

## 2. Results

### 2.1. Oral Biofilm Growth

**Figure 1:**
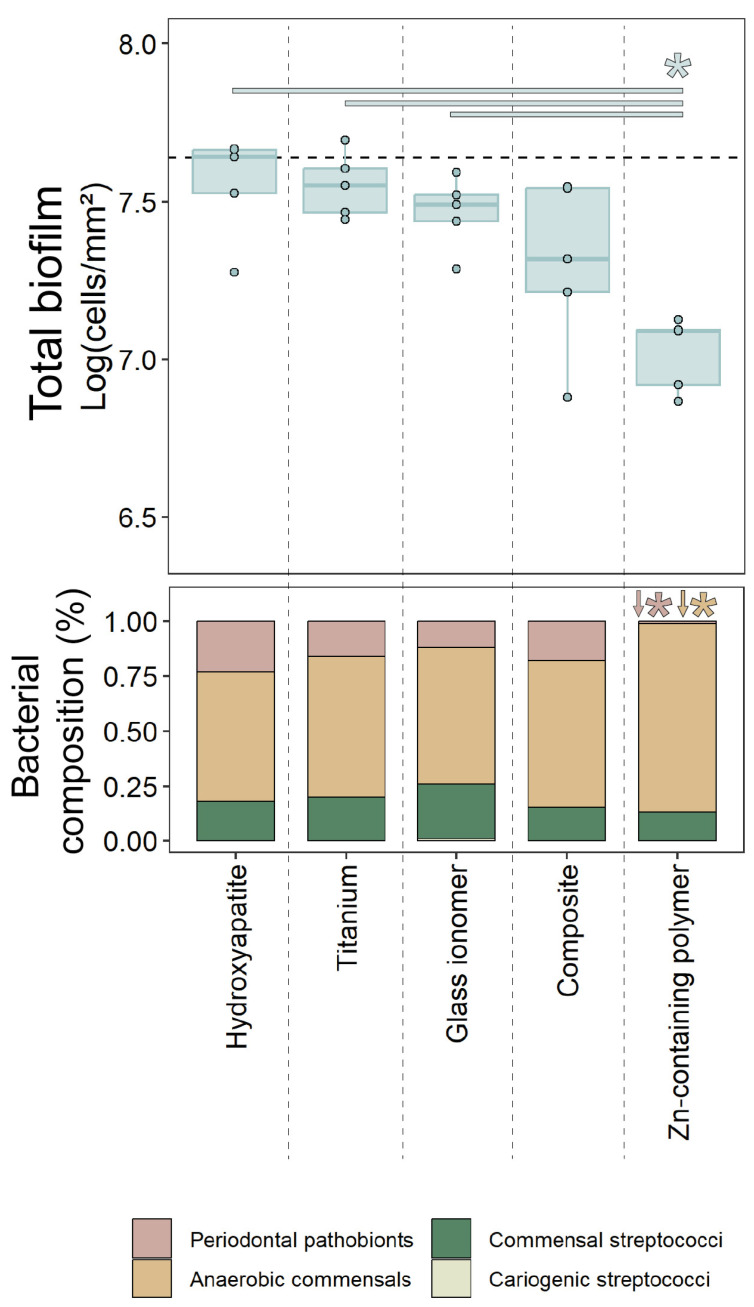
Oral multispecies biofilm growth on the investigated materials. (**Upper Panel**) Total biofilm grown after 48 h on the different biomaterials. Data are expressed as the log total of bacteria recovered per square centimeter on the disks as measured by v-qPCR (viability quantitative polymerase chain reaction). Significant differences in total biofilm formed (log(cells/mm^2^); *p* < 0.05; ANOVA with Tukey HSD; n = 5) are indicated with an asterisk above the bars combining conditions that differ significantly from each other. The black horizontal dashed line indicates the median of the hydroxyapatite control. (**Lower Panel**) Bacterial composition for the different bacterial groups in proportion to the total biofilm load. Significant differences from the control condition, hydroxyapatite disks, mimicking dentition (*p* < 0.05; ANOVA with Tukey HSD; n = 5), are presented with an asterisk in the color of the bacterial group with an arrow indicating an increase or decrease. Cariogenic streptococci represented less than 1% of the biofilm volume and were thus not visible.

Biofilm formation was evaluated on two chairside materials commonly used for custom healing abutments (glass ionomer and composite), the standard abutment material titanium, the novel Zn-containing polymer, and a hydroxyapatite control surface, mimicking dentition. The results are presented in Figure 1.

The composite material exhibited a trend toward reduced total biofilm formation compared to the control surface, though this reduction was not statistically significant (*p* = 0.15). In contrast, the Zn-containing polymer demonstrated a statistically significant reduction in total biofilm formation compared to hydroxyapatite, titanium, and glass ionomer surfaces (*p* < 0.05).

Regarding biofilm composition, biofilms on the Zn-containing polymer displayed significant differences from the other materials. While cariogenic streptococci were detected on all surfaces, they consistently comprised less than 1% of the total biofilm, with no statistically significant differences observed between the materials. For the other three bacterial groups (periodontal pathobionts, anaerobic commensals, and commensal streptococci), the proportional bacterial composition on titanium, composite, and glass ionomer surfaces were not statistically different from those on hydroxyapatite (control). However, the Zn-containing polymer surface had a significantly lower proportion of periodontal pathobionts (~25% on other surfaces vs. <1% on the polymer; *p* < 0.01), with a corresponding significant increase in anaerobic commensals (*p* < 0.01). The proportion of commensal streptococci on the Zn-containing polymer was not significantly different from that observed on the other materials.

### 2.2. Cell Adhesion and Viability

**Figure 2:**
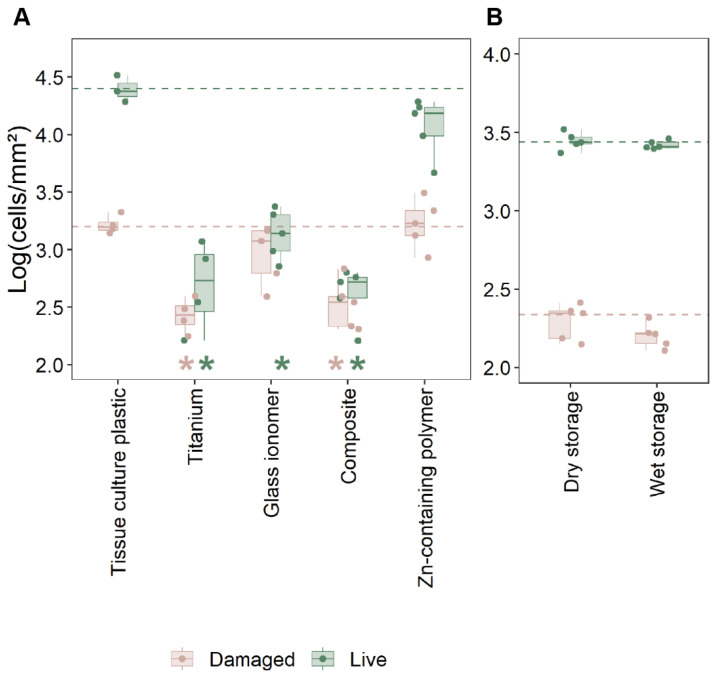
Human oral keratinocyte adhesion to investigated materials. (**A**) Live and damaged HOK-18A keratinocytes recovered after 24 h from three pooled disks as measured through flow cytometry with Hoechst and propidium iodine dual stain (Supplementary Figure S1). Significant differences from the control condition, the tissue culture plastic (*p* < 0.05; ANOVA with Tukey HSD; n ≥ 4), are presented with an asterisk in the respective color of live or damaged cells. (**B**) Adhesion of HOK-18A to the Zn-containing polymer material after dry and wet storage for 31 days. No significant differences were observed (*t*-test; *p* < 0.05; n = 5).

The adhesion of oral keratinocytes (HOK-18A) and their membrane integrity were evaluated to assess the biocompatibility of the novel Zn-containing polymer in comparison to other materials (Figure 2A and Supplementary Figure S1). Tissue culture polystyrene, known for optimal keratinocyte growth, served as the reference material since hydroxyapatite did not provide sufficient cell adhesion.

**Figure 3:**
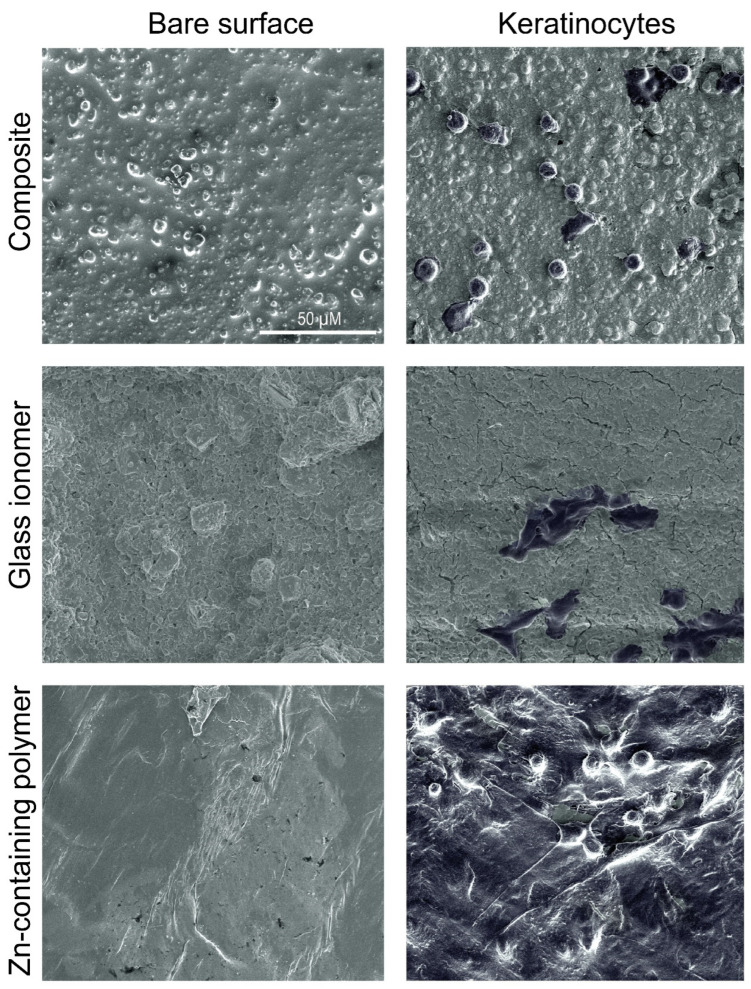
Scanning electron microscopy images of the adhesion of keratinocytes to composite, glass ionomer, and Zn-containing polymer.

Significantly fewer keratinocytes (>1 Log_10_) adhered to titanium, glass ionomer, and composite surfaces compared to the tissue culture polystyrene surface (*p* < 0.05, Figure 2A). In contrast, the Zn-containing polymer demonstrated robust cell adhesion, comparable to the polystyrene control, with no significant difference between both materials. These data were confirmed by SEM imaging (Figure 3). HOK-18A cells were observed to form monolayers on the Zn-containing polymer, a characteristic not observed on the glass ionomer or composite materials, where cells adhered more loosely (Figure 3).

Cell membrane integrity analysis revealed no statistical differences between live and damaged cells between the Zn-containing polymer and the tissue culture plastic (polystyrene) control (Figure 2A). The Zn-containing polymer supported a similar proportion of live cells. These data indicate that there is no aberrant cytotoxicity after adhesion and confirm its compatibility with cell adhesion and viability. All other materials showed a significantly lower ratio between live and damaged epithelial cells, with no significant difference between these three materials.

In order to confirm that the biocompatibility of the Zn-containing polymer was not lost over time or influenced by storage conditions, the material was evaluated after 31 days of storage under wet and dry conditions (Figure 2B). No significant differences in cell adhesion were observed between the two conditions, indicating that the material retained its ability to support good cell adhesion over time. These findings, combined with data on file showing no detectable leaching of zinc, suggest that the material’s favorable properties are inherent and remain consistent.

### 2.3. Cytotoxicity of Materials

**Figure 4:**
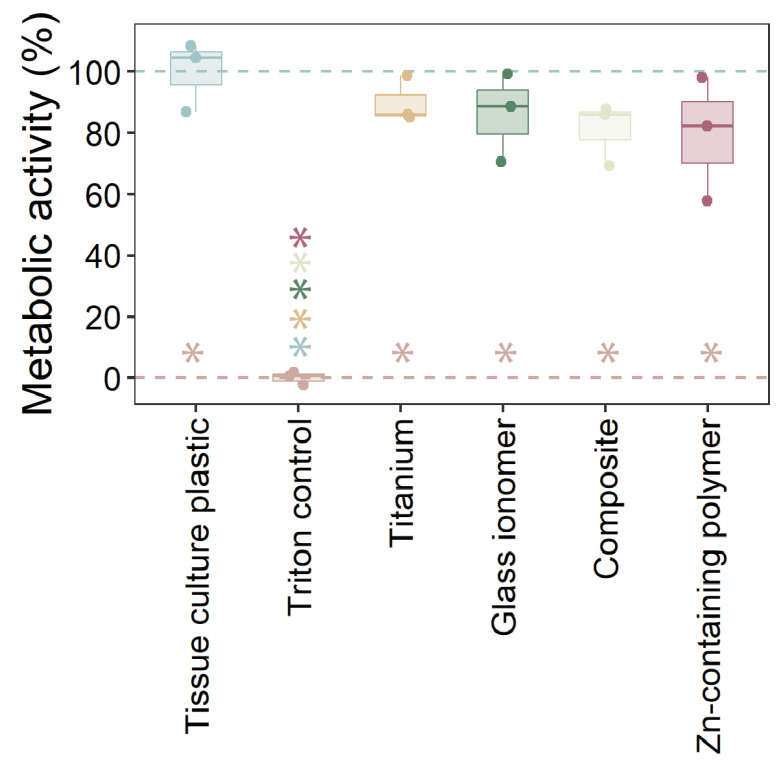
The cytotoxicity of tested materials to human oral keratinocytes. The cytotoxicity of the materials on HOK-18A cells after 24 h of co-incubation, measured through metabolic activity via XTT-assay. The data are presented as the metabolic activity relative to the tissue culture plate control. Significant differences (*p* < 0.05; ANOVA with Tukey HSD; n = 3) are presented with an asterisk in the respective color of the condition.

The cytotoxicity of the tested materials on HOK-18A cells was assessed by measuring metabolic activity (Figure 4). As expected, the Triton X-100 positive control displayed complete cytotoxicity, resulting in minimal metabolic activity. In contrast, none of the tested materials caused a statistically significant reduction in metabolic activity compared to the tissue culture polystyrene. However, all materials exhibited slightly lower metabolic activity levels than cells cultured on tissue culture polystyrene, which serves as the optimal reference surface for cell growth. These results indicate that the tested materials, including the Zn-containing polymer, do not exhibit cytotoxic effects on HOK-18A cells and maintain acceptable biocompatibility.

## 3. Discussion

This study investigated the potential of a novel thermoplastic Zn-containing polymer for use in chairside custom healing abutments, focusing on its biocompatibility, cell growth support, and antimicrobial properties. The main findings revealed that the Zn-containing polymer outperformed traditional materials such as titanium, glass ionomer, and composite in several critical areas, including biofilm development, keratinocyte adhesion, and cytotoxicity. Additionally, the material’s favorable properties were maintained under different storage conditions, highlighting its stability and suitability for long-term clinical use.

**Figure 5:**
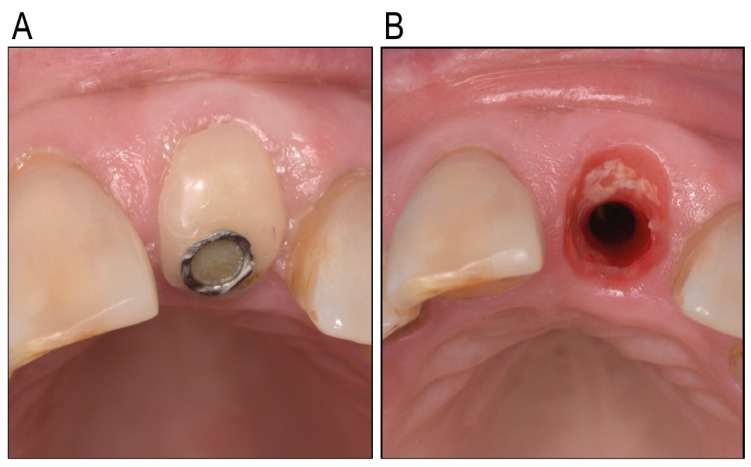
Clinical illustration of a composite individualized healing abutment, in situ for 14 days (**A**) and the related soft-tissue reaction (**B**). Inflammation is clearly visible. Informed consent was acquired from patient 89668917 @UZ Leuven.

Customized healing abutments for oral implants are becoming more popular, given their value in immediate peri-implant soft-tissue engineering. The key required properties for using such an abutment are biocompatibility with human tissues, antibacterial activity, and ease of use. Soft tissue attachment to the abutment is a critical determinant of peri-implant tissue health, acting as a biological seal that protects underlying tissues from bacterial invasion and inflammation, and potentially maintaining the soft tissue profile. The findings of this study emphasize the importance of material selection for healing abutments in promoting this attachment. The Zn-containing polymer demonstrated superior biocompatibility compared to traditional materials, such as titanium, glass ionomer, and composite, which exhibited significantly lower keratinocyte adhesion and a higher proportion of damaged cells, which was clinically observed as inflammation around the individualized healing abutment (Figure 5). Unlike these materials, the polymer supported the formation of stable keratinocyte monolayers, a structural organization that is vital for maintaining the functional integrity of the epithelial barrier. This feature underscores the polymer’s potential to enhance peri-implant healing and maintain tissue stability. The observed biocompatibility of the Zn-containing polymer is likely influenced by its surface properties, which play a pivotal role in cellular attachment and proliferation [30]. Zinc’s incorporation into the polymer matrix further enhances these effects.

Zinc-modified biomaterials have been shown to influence the proliferation and adhesion of various cell types, including epithelial cells like keratinocytes [31,32,33]. Zinc ions can influence cell behavior by modulating gene expression related to cell adhesion, growth, and angiogenesis. They can also activate signaling pathways that promote cell proliferation and differentiation [34,35].

These properties are critical for applications requiring robust epithelial barriers, such as wound healing, infection prevention, and the maintenance of peri-implant tissue morphology.

The cytotoxicity results confirmed the safety of the Zn-containing polymer, with no significant reductions in metabolic activity compared to the control. This is a critical consideration for materials that come into direct contact with gingival tissues. Traditional materials, such as composites and glass ionomers, have raised concerns due to the potential release of cytotoxic components, including monomers like UDMA and TEGDMA [36]. The absence of these harmful monomers in the Zn-containing polymer positions it as a safer alternative for use in custom healing abutments. Additionally, the polymer retained its biocompatibility after 31 days of wet or dry storage, highlighting its long-term stability and reliability for clinical applications.

The Zn-containing polymer also demonstrated a significant reduction in total biofilm formation and a notable shift in biofilm composition toward a less pathogenic profile compared to traditional materials and hydroxyapatite, a commonly used control surface to mimic dentition. Specifically, the polymer exhibited a lower proportion of periodontal pathobionts and a corresponding increase in anaerobic commensals. This favorable modulation of the local microbial environment highlights the polymer’s potential to reduce pathogenic colonization and lower the risk of peri-implant infections. Such antimicrobial modulation was not observed for the other tested materials. These effects are likely attributable to the antimicrobial and anti-biofilm properties of zinc, which is incorporated into the polymer matrix. Zinc has well-documented broad-spectrum antimicrobial activity, primarily through its ability to disrupt microbial cell membranes, causing cell lysis and death [37]. In addition, zinc has been shown to inhibit biofilm formation in various bacterial species, including Staphylococcus aureus and Escherichia coli, by interfering with quorum sensing and other signaling pathways essential for biofilm development [38]. These findings align with the present study, where the Zn-containing polymer showed superior performance in minimizing biofilm formation, even in a complex multispecies oral biofilm model. Moreover, zinc’s ability to modulate the microbial environment to favor less pathogenic bacterial communities has been previously demonstrated [39]. This property was evident in our results, where the Zn-containing polymer consistently reduced periodontal pathobionts and promoted the growth of anaerobic commensals, setting it apart from other materials. Traditional materials, while biocompatible, lack such antimicrobial and biofilm-modulating properties, leaving the peri-implant environment more susceptible to pathogenic colonization and associated complications. In contrast, the dual-action effect of the Zn-containing polymer—its ability to inhibit biofilm formation and favor a more benign microbial community—positions it as a promising material for enhancing peri-implant tissue health and reducing biofilm-associated complications.

The findings of this study have significant implications for implant dentistry. The Zn-containing polymer’s ability to promote keratinocyte adhesion, reduce biofilm formation, and maintain biocompatibility over time suggests that it could improve peri-implant tissue health and stability. These properties are particularly relevant in preventing peri-implant complications, such as soft tissue inflammation and collapse. What is of particular interest is the fact that the zinc is not being released or leaching out of the polymer. Additionally, the polymer’s antimicrobial properties may reduce the risk of bacterial colonization during the critical healing phase, supporting faster and more predictable soft tissue maturation. This could lead to better long-term outcomes for implant-supported restorations, including improved esthetic and functional results. Additionally, the Zn-containing polymer holds significant promise for new biomedical applications such as wound healing dressings and tissue engineering scaffolds [21,40,41,42,43], in particular within the dental field.

While the in vitro results are promising, further studies are needed to translate and validate the in vitro results to the clinical performance of the thermoplastic Zn-containing polymer for chairside custom healing abutments. The oral cavity presents a dynamic environment where factors such as saliva flow, enzymatic activity, and host immune responses can influence material performance [44]. Saliva can dilute or inactivate antimicrobial agents of the Zn-containing polymer, while innate and adaptive immune responses may alter host–material interactions over time. While the Zn-containing polymer disrupted the pathogenic bacteria without affecting beneficial species in the biofilm model, actual in vivo oral microbiomes are complex and potentially more resilient [45]. Long-term in vivo studies should evaluate its impact on peri-implant tissue health, patient comfort, and soft tissue stability. Additionally, mechanistic studies exploring how zinc influences cellular behavior and biofilm dynamics could provide deeper insights into its therapeutic potential. Future research should also investigate the polymer’s performance under different clinical scenarios, such as immediate implant placement or cases with compromised soft tissue conditions.

Another limitation of this study is that PEEK was not used as a material. This is because PEEK was not considered as a chairside custom healing abutment material. This study primarily focused on custom healing abutment materials that can be used chairside. Titanium was used as the standard abutment material.

## 4. Materials and Methods

### 4.1. Investigated Materials

**Figure 6:**
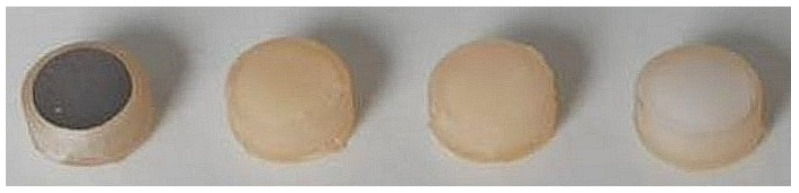
Investigated biomaterials: titanium, glass ionomer, composite, and Zn-containing polymer.

Four different test materials were investigated: commercially pure titanium (grade 2, Salomon’s Metalen, Groningen, The Netherlands; prepared according to Aktan et al. [46]), glass ionomer (Fuji™ II LC; GC, Haasrode, Belgium), composite (Filtek™ Supreme; 3M, Diegem, Belgium), and a Zn-containing polymer (Oral Surgical Granulate^®^, Elemental, Belgium) (Figure 6). The samples were fabricated as cylinders with dimensions of 5 mm in diameter and 2 mm in thickness using molds printed with dental resin (Formlabs, Sommervile, MA, USA). Each material was cured according to the manufacturer’s instructions. Hydroxyapatite (Himed, Old Bethpage, NY, USA) and tissue culture polystyrene (Sarstedt, Nümbrecht, Germany) functioned as controls for biofilm growth on dentition and the optimal growth survival of keratinocytes, respectively.

In order to evaluate the stability of the Zn-containing polymer in the oral cavity, the polymer was stored either dry at room temperature or in phosphate-buffered saline at 37 °C for 31 days, both in the dark.

### 4.2. Bacterial and Biofilm Growth on the Materials

The multispecies biofilm formation with oral bacteria on the different materials was performed using a model previously described by Slomka et al. [47] and Van Holm et al. [48]. Oral bacteria (Table 1) were maintained on blood agar aerobically for A. actinomycetemcomitans and streptococci, and anaerobically for the other species before a multispecies biofilm seed culture was created. Briefly, a multispecies community of 14 oral bacteria was established in a bioreactor in modified BHI (BHI-2, enriched with mucin, yeast extract, glutamate, cysteine, hemine, and menadione [47]), and after stabilization, a biofilm seed culture was sampled (1 × 10^8^ cells/mL, evaluated with flow cytometry [49]) and biofilms were formed in BHI-2 on the investigated materials (titanium, glass ionomer, composite, freshly molded Zn-containing polymer, and hydroxyapatite as a mimic for dental surfaces) for 24 h micro-aerophilically (6% oxygen) at 37 °C. Cells were thereafter recovered from the biofilms enzymatically with trypsin (45 min) and mechanically by vortexing (30 s), and subsequently centrifuged and resuspended in a final volume of 0.5 mL of PBS. Then, 90 µL of this cell suspension was viability-treated with PMAxx [50] (Biotium, Hayward, CA, USA) and extracted with the QIAamp^®^ DNA Mini Kit (QIAGEN, Hilden, Germany) to a final volume of 200 µL per sample. Taking dilutions into account, DNA was analyzed through qPCR according to Herrero et al. [51], with the use of revised sequences of the primers for amplicons to above 150 base pairs. The primer and probe sequences and qPCR assay conditions are listed in Supplementary Table S1.

### 4.3. Epithelial Cells

Human oral keratinocytes (HOK-18A; immortalized HOK with recombinant HPV-18 by [52] and handled according to [53]) were used as a cell line and were grown at 37 °C with 5% CO_2_ with Keratinocyte SFM (Thermo Fisher Scientific, Waltham, MA, USA) supplemented with epidermal growth factor and bovine pituitary extract, according to the manufacturer’s instructions.

### 4.4. Cell Adhesion to Materials—Cell Viability

To evaluate the growth of human cells to the described materials (titanium, glass ionomer, composite, freshly molded Zn-containing polymer, 31-day dry-stored Zn-containing polymer, and 31-day wet-stored Zn-containing polymer) and the compatibility of the materials through cell membrane integrity analysis, human oral keratinocytes (HOK-18A) were seeded at 10^5^ cells/mL to the test materials and to an empty polystyrene well (tissue culture plastic as positive control for optimal cell growth instead of hydroxyapatite) to adhere and form monolayers over 24 h. Afterwards, three disks per material were pooled and treated with 500 µL 0.05% trypsin to recover the adhered HOK-18A. The cells were then stained with a final concentration of 1 µg/mL Hoechst (BD Biosciences, New Jersey, USA) and 20 µM propidium iodine (Thermo Fisher Scientific, Massachusetts, USA) for 20 min, analyzed through flow cytometry with a FACSVerse cytometer with a blue 488 nm laser and a red 640 nm laser (BD Biosciences, Franklin Lakes, NJ, USA), with a flow sensor to enumerate cells (events/µL), and recalculated to the surface area of the disks (cells/mm^2^). Forward scatter (FSC-A), side scatter (SSC-A), green fluorescence signals (FITC-A), and red fluorescence signals (PerCP-CY5.5-A) were used to characterize healthy HOK-18A cultures via gating from the control conditions with an inherent low red signal (Supplementary Figure S1; low PerCP-CY5.5A signal due to low propidium iodine entering the cells with intact membranes).

In parallel, keratinocytes attached to the materials were fixated for scanning electron microscopy with a 2.5% glutaraldehyde solution in cacodylate buffer (0.1 M pH 7.4) for 30 min and then dehydrated (ethanol exposures 30, 50, 70, 90, and 3 × 100% for 20 min) [46]. After drying, the disks were sputter-coated with a 5 nm Pt coating (Q150T ES plus, Quorum tech, East Sussex, UK). Using a Nova NanoSEM 450 (FEI, Hillsboro, OR, USA), and representative images were taken in high-vacuum settings with a high-resolution in-lens (magnetic immersion lens) at 5 keV acceleration voltage, with a working distance of 5 mm and a spot size of 3.

### 4.5. Cytotoxicity of Materials—Metabolic Activity

To evaluate the cytotoxicity of the materials (titanium, glass ionomer, composite, and freshly molded Zn-containing polymer), HOK-18A keratinocytes were grown confluently on tissue culture plates. An orthodontic elastic band was used as a spacer on which the materials were placed. After 24 h of incubation, the materials and spacers were removed and the metabolic activity of the cells was evaluated with an XTT-assay (Roche, Basel, Switzerland) according to the manufacturer’s instructions, with 1% Triton X-100 used as positive cytotoxic control.

### 4.6. Statistical Analysis

Statistical analysis was performed in R 4.2.0. All data were normally distributed (Shapiro–Wilk test) and groups displayed equal variation (Levene’s test). All statistical differences between conditions were analyzed with one-way ANOVA with Tukey HSD multiple comparisons (95% CI, significance at *p* < 0.05). For the data of the two groups in Figure 2B, Student’s *t*-test was performed.

## 5. Conclusions

In conclusion, this study highlights the potential of a novel thermoplastic Zn-containing polymer for use in chairside custom healing abutments. Its combination of biocompatibility, antimicrobial properties, and practicality for chairside use addresses many limitations of traditional materials. By supporting healthy peri-implant tissues and reducing the risk of complications, this material might represent a significant advancement in implant dentistry. With further clinical validation, the Zn-containing polymer could become a valuable tool for improving patient outcomes and enhancing the predictability of soft tissue support in immediate implant and other implant placement procedures.

## Figures and Tables

**Table 1:** Oral bacteria in the biofilm model.

Group	Name
Periodontal pathobionts	*Aggregatibacter actinomycetemcomitans* ATCC 43718
	*Prevotella intermedia* ATCC 25611
	*Porphyromonas gingivalis* ATCC 33277
	*Fusobacterium nucleatum* ATCC 20482
Anaerobic commensals	*Actinomyces viscosus* DSM 43327
	*Actinomyces naeslundii* ATCC 51655
	*Veillonella parvula* DSM 2008
Cariogenic streptococci	*Streptococcus mutans* ATCC 20523
	*Streptococcus sobrinus* ATCC 20742
Commensal streptococci	*Streptococcus oralis* DSM 20627
	*Streptococcus sanguinis* LM14657
	*Streptococcus gordonii* ATCC 49818
	*Streptococcus mitis* DSM 12643
	*Streptococcus salivarius* TOVE-R

## Data Availability

The data that support the findings of this study are available from the corresponding author upon reasonable request.

## Supplementary Materials

The following supporting information can be downloaded at: https://www.mdpi.com/article/10.3390/antibiotics14050441/s1, Figure S1: Example flow cytometric data and gates of each condition of Figure 2; Table S1: Primers and qPCR conditions for v-qPCR.

## Abbreviations

The following abbreviations are used in this manuscript:

Zn	Zinc
HOK-18A	Human oral keratinocyte cell line
SSA	Socket seal abutments
v-qPCR	Viability quantitative polymerase chain reaction
