# Supplementary material for: Thermoplastic Zinc-Infused Polymer for Chairside Socket Seal Abutments Enhances Antimicrobial and Tissue-Integrative Properties
Source: Antibiotics (Basel). 2025 Apr 27;14(5):441. doi: 10.3390/antibiotics14050441 (PMC12108465; doi:10.3390/antibiotics14050441)
Supplement: Supplementary file 1 [file antibiotics-14-00441-s001.zip › antibiotics-3575461-supplementary.pdf]

Supplementary information

Supplementary table S1: Primers & qPCR conditions for v-qPCR. qPCR was performed according to Van Holm et al. with a CFX96 real-time system (Bio-Rad, Hercules, CA, USA) with reactions consisting of 12.5 mL of Takyon Rox probe master mix dTTP blue (Eurogentec, Seraing, Belgium), 1 mL of each primer (IDT, Haasrode, Belgium) and probe (all DD probes, 5'-FAM [6-carboxyfluorescein] and 3'-TAMRA [6-carboxytetramethylrhodamine]; Eurogentec, Seraing, Belgium) and 4.5 mL of Milli-Q water. Cycle conditions consisted of an initial step at 50°C for 2 min and 95°C for 10 min, followed by 45 cycles of 95°C for 15 s and 60°C for 1 min.

Species	Primers & Probe (Final concentrations)		Amplicon length (bp)	Target
Aggregatibacter actino-mycetemcomitans	Foward	CGG TGT CGA TTT GGG GAT TGG (300 nM)	237	16S rRNA gene
	Reverse	TGC AGC ACC TGT CTC AAA GC (300 nM)		
	Probe	AGA ACT CAG AGA TGG GTT TGT GCC TTA GGG (100 nM)		
Prevotella intermedia	Forward	TGT GCC CYT TTG CAT TTA CCC TTC (300 nM)	216	16S rRNA gene
	Reverse	CAC CAT GAA TTC CGC ATA CG (900 nM)		
	Probe	TGG CGG ACT TGA GTG CAC GC (200 nM)		
Porphyromonas gingivalis	Forward	CCG TAA GAA TAA GCA TCG GCT AAC TC (300 nM)	195	16S rRNA gene
	Reverse	CAC GAA TTC CGC CTG C (300 nM)		
	Probe	CAC TGA ACT CAA GCC CGG CAG TTT CAA (100 nM)		
Fusobacterium nucleatum	Forward	GGA TTT ATT GGG CGT AAA GC (300 nM)	191	16S rRNA gene
	Reverse	ATC TGT CCA GTA AGC TGG CTT CC (300 nM)		
	Probe	CTC TAC ACT TGT AGT TCC G (300 nM)		
Streptococcus mutans	Forward	GCC TAC AGC TCA GAG ATG CTA TTC T (900 nM)	114	gtfB gene
	Reverse	GCC ATA CAC CAC TCA TGA ATT GA (900 nM)		
	Probe	TGG AAA TGA CGG TCG CCG TTA TGA A (100 nM)		
Streptococcus sobrinus	Forward	AAA TAC GGC CAG TGC CAA AG (200 nM)	165	gtfT gene
	Reverse	CCA GCC TGA GAT TCA GCT TGT (200 nM)		
	Probe	CCT GCT CCA GCG ACA AAG GCA GC (250 nM)		
Actinomyces naeslundii	Forward	TCG AAA CTC AGC AAG TAG CCG (200 nM)	96	gene encoding unknown protein
	Reverse	AGA GGA GGG CCA CAA AAG AAA (200 nM)		
	Probe	GGG TAC TCT AGT CCA AAC TGG CGG ATA GCG (100 nM)		
Actinomyces viscosus	Forward	GTG AAG GAG CCA GCT TGC TGG TTC TG (200 nM)	155	16S rRNA gene
	Reverse	CGG AAC AAA CCT TTC CCA GGC (200 nM)		
	Probe	ATG AGT GGC GAA CGG GTG AGT AAC (125 nM)		
Veillonella parvula	Forward	GAC GAA AGT CTG ACG GAG CA (200 nM)	171	16S rRNA gene
	Reverse	TGC CAC CTA CGT ATT ACC GC (200 nM)		
	Probe	AGC TCT GTT AAT CGG GAC GAA AGG C (125 nM)		
Streptococcus oralis	Forward	ACC AGC AGA TAC GAA AGA AGC AT (400 nM)	229	*gtfR* gene
	Reverse	AGG TTC GGG CAA GCG ATC TTT CT (400 nM)		
	Probe	AAG GCT GCT GTT GCT GAA GAA GT (100 nM)		
Streptococcus sanguinis	Forward	CAA AAT TGT TGC AAA TCC AAA GG (600 nM)	75	*gtfP* gene
	Reverse	GCT ATC GCT CCC TGT CTT TGA (600 nM)		
	Probe	AAA GAA AGA TCG CTT GCC AGA ACC GG (100 nM)		
Streptococcus gordonii	Forward	GAA GAA CTG GGT AGC GAT TGC T (400 nM)	262	*gtfG* gene
	Reverse	GTT AGC TGT TGG ATT GGT TGC C (400 nM)		
	Probe	AGA ACA GTC CGC TGT TCA GAG CAA (100 nM)		
Streptococcus mitis	Forward	GGC TCG TAG TCT GGA GAT GG (600 nM)	133	16S rRNA gene
	Reverse	TAG GTC GTC GTC CCA AGG AA (600 nM)		
	Probe	CGA AGA GCA CCA ATA GCA CCT CCC (140 nM)		
Streptococcus salivarius	Forward	GAC GAT GAC TGT CAA CTT GAC AC (400 nM)	247	*Dextranase* gene
	Reverse	ACC GTA ACG TGG GAA AAC TG (400 nM)		
	Probe	GTA GCG TCA GAG TGG TTG AC (100 nM)		

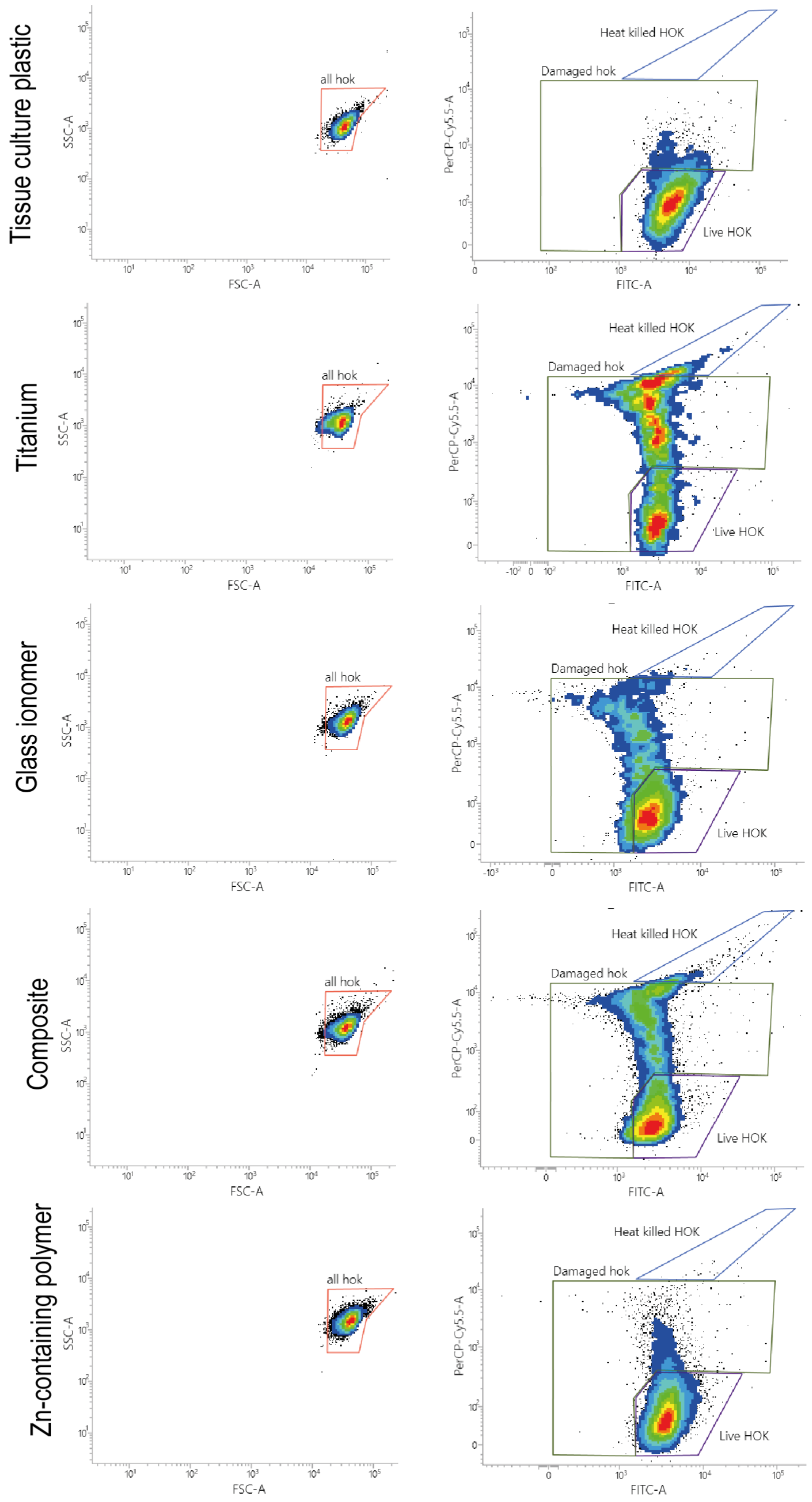

Supplementary figure S1: Example flow cytometric data and gates of each condition of figure 3. Events within live and damaged gates were enumerated with the flow sensor (events/μL).
